# A temporal comparison of sex-aggregation pheromone gland content and dynamics of release in three members of the *Lutzomyia longipalpis* (Diptera: Psychodidae) species complex

**DOI:** 10.1371/journal.pntd.0006071

**Published:** 2017-12-01

**Authors:** Mikel A. González, Krishna K. Bandi, Melissa J. Bell, Reginaldo P. Brazil, Erin Dilger, Angel Guerrero, Orin Courtenay, James G. C. Hamilton

**Affiliations:** 1 Division of Biomedical and Life Sciences, Faculty of Health and Medicine, Lancaster University, Lancashire, United Kingdom; 2 Laboratório de Doenças Parasitárias, Instituto Oswaldo Cruz, FIOCRUZ, Rio de Janeiro, Brazil; 3 School of Life Sciences, University of Warwick, Coventry, United Kingdom; 4 Department of Biological Chemistry and Molecular Modelling, IQAC (CSIC), Barcelona, Spain; Universidade Federal do Rio de Janeiro, BRAZIL

## Abstract

**Background:**

*Lutzomyia longipalpis* is the South American vector of *Leishmania infantum*, the etiologic agent of visceral leishmaniasis (VL). Male *L*. *longipalpis* produce a sex-aggregation pheromone that is critical in mating, yet very little is known about its accumulation over time or factors involved in release. This laboratory study aimed to compare accumulation of pheromone over time and determine factors that might influence release in three members of the *L*. *longipalpis* species complex.

**Methodology/Principal findings:**

We investigated male sex-aggregation pheromone gland content at different ages and the release rate of pheromone in the presence or absence of females under different light conditions by gas chromatography-mass spectrometry (GC-MS). Pheromone gland content was determined by extraction of whole males and pheromone release rate was determined by collection of headspace volatiles. Pheromone gland content appeared age-related and pheromone began to accumulate between 6 to 12 h post eclosion and gradually increased until males were 7–9 days old. The greatest amount was detected in 9-day old Campo Grande males ((*S*)-9-methylgermacrene-B; *X* ± SE: 203.5 ± 57.4 ng/male) followed by Sobral 2S males (diterpene; 199.9 ± 34.3) and Jacobina males ((*1S*,*3S*,*7R*)-3-methyl-α-himachalene; 128.8 ± 30.3) at 7 days old. Pheromone release was not continuous over time. During a 4-hour period, the greatest quantities of pheromone were released during the first hour, when wing beating activity was most intense. It was then substantially diminished for the remainder of the time. During a 24 h period, 4–5 day old male sand flies released approximately 63 ± 11% of the pheromone content of their glands, depending on the chemotype. The presence of females significantly increased pheromone release rate. The light regime under which the sand flies were held had little influence on pheromone release except on Sobral 2S chemotype.

**Conclusions/Significance:**

Accumulation of pheromone appears to occur at different rates in the different chemotypes examined and results in differing amounts being present in glands over time. Release of accumulated pheromone is not passive, but depends on biotic (presence of females) and abiotic (light) circumstances. There are marked differences in content and release between the members of the complex suggesting important behavioural, biosynthetic and ecological differences between them.

## Author summary

The Dipteran subfamily Phlebotominae includes the genera *Lutzomyia* and *Phlebotomus* among which several species are important vectors of parasitic and bacterial pathogens. The sand fly *Lutzomyia longipalpis* is considered the main vector of visceral leishmaniasis (VL) in the New World. Based on the main component of the male sex-aggregation pheromone gland, different sex pheromone-producing populations (chemotypes) of *L*. *longipalpis* are recognized in Brazil. Given the importance of the sex-aggregation pheromones in the biology of this species complex, we present here the first attempt to study how pheromone accumulates in the glands over time and factors that might influence its release in the three most common chemotypes from Brazil. Our results demonstrated that pheromone first starts to accumulate a few hours post-eclosion (6–12 h) and this continues over 15 days. Pheromone release is a dynamic process which varies between the 3 chemotypes depending on biotic factors, such as light regime and presence/absence of conspecific females. This work provides valuable information, critical to our understanding of the behaviour and ecology of *L*. *longipalpis* sand flies and which will contribute to investigations to improve field-based pheromone control and monitoring of *L*. *longipalpis* sand flies.

## Introduction

There are over 800 known phlebotomine sand fly species, but only approximately 56 *Lutzomyia and Phlebotomus* species are proven or suspected vectors of human leishmaniasis [1]. Among them, *L*. *longipalpis* is the primary vector of *Leishmania infantum*, the etiological agent of visceral leishmaniasis (VL) in the Americas [2]. The presence of *L*. *longipalpis* has been recorded in 12 countries, including Argentina, Bolivia, Colombia, Costa Rica, El Salvador, Guatemala, Honduras, Mexico, Nicaragua, Paraguay, and Venezuela. It is also widely distributed throughout Brazil [1]. During the last two decades it has colonised urban environments and expanded its geographical range, which has resulted in an increase in the number of cases of canine and human VL [3, 4].

The taxonomic status of *L*. *longipalpis* has been uncertain since it was first described by Mangabeira in 1969 [5] and recent analysis of molecular and genetic markers [6], morphological features [7], copulation songs [8], and chemical communication [9] all indicate that *L*. *longipalpis* is a complex of recently evolved cryptic species [10]. However, there is no consensus on the number of species in the complex or their geographic distributions [7, 11, 12].

In many Dipteran species, sex pheromones, together with visual, tactile and acoustic signals, play an important role in courtship behaviour [13]. Sex-aggregation pheromones occur in male *L*. *longipalpis* [9, 14] and may be widespread in the genus *Lutzomyia*. There is chemical evidence that they also occur in *L*. *cruzi* [15], *L*. *pseudolongipalpis* [16], *L*. *pessoai* [17], *L*. *lichyi* [18], *L*. *lenti*, *L*. *carmelinoi* [19] and *L*. *cruciata* [20]. They have also been found in the closely related genus *Sergentomyia*, e.g. *S*. *minuta* and *S*. *fallax* [21]. These species produce volatile terpenoid compounds that are structurally similar to the sex-aggregation pheromones of the *L*. *longipalpis* species complex. Based on behavioural experiments, there is some evidence that pheromones may also play a role in the mating of *Phlebotomus papatasi* [22], the main vector of the Old World cutaneous leishmaniasis [1].

In the *L*. *longipalpis* species complex the sex-aggregation pheromones have been studied for both their taxonomic value and to exploit their vector control potential [23]. Analysis of the main terpene component of the sex-aggregation pheromone gland extract has shown that there are at least four distinct chemotypes of *L*. *longipalpis* [9, 24]: i) (*1S*,*3S*,*7R*)-3-methyl-α-himachalene (3MαH), a novel bicyclic methylsesquiterpene with a 16 carbon skeleton (C16, molecular weight (mw): 218). In Brazil, this chemotype has been found only in Bahia State; ii) (*S*)-9-methylgermacrene-B (9MGB), a novel monocyclic methylsesquiterpene (C16, mw: 218). It is also the most widespread chemotype in the Americas, and is found in Argentina, Colombia, Paraguay, Venezuela, Honduras, Costa Rica and Brazil. This chemotype is common in Brazil, particularly in the centre, south and to a lesser extent, in the northeast of the country; iii) a partially characterised diterpene (C20, mw: 272) is the second most widely distributed chemotype in Brazil [25]. It is mainly found in the northeastern states although recent reports indicate that it is present in the southeast [4]; iv) a related partially characterised diterpene has also been found only in specimens from Jaíba, Minas Gerais State, Brazil [25].

A racemic version (containing both the *R* and *S* isomers) of the 9-methylgermacrene-B sex pheromone has been synthesised in bulk, shown to be active in the field and formulated for long-term controlled release [23]. It is currently being evaluated for its potential to reduce the risk of canine VL infection in a lure-and-kill vector control tool and for enhanced monitoring. Optimising the controlled release formulation of the synthetic pheromone for both of these functions relies on knowledge of how pheromone is produced and released by individuals and groups of males under different conditions.

The sex-aggregation pheromone is synthesised and stored in glandular tissue underlying the abdominal tergites and is transported via cuticular ducts to modified structures, “papules”, on the cuticle surface [19, 26]. Pheromone glands in young, 0–6 h old, males are undifferentiated but appear to be fully differentiated in 4-day old males [27]. Pheromone production is currently believed to start after 12 h and increase continuously for 3 days when it reaches a plateau [28].

No studies have been undertaken to determine if differences in pheromone gland content occur between different members of the species complex nor to determine which factors, if any, might contribute to subsequent pheromone release. It has been suggested that wing-fanning, which occurs in males during courtship, may help to distribute pheromone [29–32] and it has also been suggested that frequent mating attempts during courtship might increase pheromone release but deplete glandular pheromone reserves [29]. Males with depleted gland contents are less successful at obtaining mating attempts than males with fuller glands [29].

In this study, we have investigated the accumulation of pheromone and the dynamics of release for each of three members of the *L*. *longipalpis* species complex. Specifically, we addressed the following questions: a) do males of different chemotypes have the same total amount of pheromone (gland content) over time? b) how much pheromone is released from the glands into the atmosphere? c) is pheromone release affected by the presence of conspecific females? and d) do light conditions have an effect on pheromone release?

## Methods

### Sand flies

All three colonies (chemotypes) of *L*. *longipalpis* used in this study were originally established from females collected using miniature CDC light traps in chicken shelters. The Jacobina (3MαH), and Campo Grande (9MGB) colonies were established from groups of females collected in Jacobina, Bahia State, (11° 11' S, 40° 31' W) and Campo Grande, Mato Grosso do Sul State (20° 28’ S, 54° 37’ W). The Sobral 2S (diterpene) colony was established from Sobral, Ceará State (3° 41’ S, 40° 20’ W).

The Jacobina colony was originally established in 1974 [33] (estimated 380^th^ generation). The Campo Grande colony was established in 2009 (estimated 63^rd^ generation) and the Sobral 2S colony was established in 2013 (estimated 27^th^ generation).

Previously, chromatographic analysis of the pheromone gland contents of individual males from both Jacobina and Campo Grande colonies established that both were allopatric and contained representatives of only one chemotype [17, 23]. In Sobral the diterpene and 9MGB chemotypes are sympatric. The diterpene chemotype males can be distinguished from the 9MGB males by the number of pale patches on the abdomen. The diterpene chemotype males have two patches (2S), whereas the 9MGB chemotype males have only 1 (1S). Using careful iso-female rearing [34] we were able to establish a diterpene producing colony. Evidence collected from the Sobral field site indicates that the 2 chemotypes do not cross-mate [9, 24].

Sobral 2S males produce a Burst-type copulatory song and the Jacobina males produce a Pulse-type copulatory song, categorised as P1 because of trains of pulses with usually two or three cycles per pulse [12]. The Campo Grande chemotype copulatory song has not yet been categorised.

The sand flies were maintained at 28 ± 2°C, 80 ± 5% relative humidity (RH) and a 12:12 light:dark (L:D) photoperiod in an insectary at Lancaster University (United Kingdom). Immature stages were maintained in rearing pots in which the bottom was filled with a layer (2 cm) of dampened Plaster of Paris to maintain humidity. Females and males were pooled together 3–4 days after eclosion in Barraud cages (18 x 18 x 18 cm) and the females were routinely blood-fed on anaesthetized mice to maintain the colony.

Sand fly blood feeding for colony maintenance was performed according to the guidelines and regulations of the Animals in Science Regulation Unit (ASRU) and in accordance with the terms of a regulated licence (PPL 40/3279) in compliance with the UK Home Office, Animals (Scientific Procedures) Act (ASPA) regulations. All procedures involving animals were reviewed and approved by the Animal Welfare and Ethical Review Board (AWERB) at Lancaster University.

### Coupled gas chromatography-mass spectrometry (GC-MS)

Analysis of male sex-aggregation pheromone gland extracts and headspace entrainment extracts was performed on an Agilent 7890A/5975C GC-MS (Agilent Technologies UK Ltd, Cheshire, UK) operating in electron impact mode. Chromatographic analysis was conducted on a non-polar HP-5MS capillary column, 30 m x 0.25 mm i.d., 0.25 μm film thickness (Agilent, UK), using H_2_ as carrier gas at 1 ml min^-1^. Samples were introduced via an on-column injector set at 40°C. The temperature program was an initial temperature (40 ^o^C) held for 2 min and then increased at 10°C min^-1^ to a final isothermal temperature (250 ^o^C) held for 10 min.

### Cleaning of glassware and entrainment equipment

To remove potential contaminants, all glassware was carefully cleaned prior to use by washing in a 10% detergent solution. It was then rinsed with distilled water, dried with acetone and finally heated in an oven at 180ºC for 12 h.

The components of the the entrainment apparatus were connected with fluorinated ethylene propylene (FEP) tubing that was cleaned internally by rinsing with hexane (BDH HiPerSolv, 97%, VWR, Lutterworth, UK) before and after each entrainment.

A new clean 50 ml r/b flask was used for every experimental replicate to ensure that the potential presence of residual pheromone did not interfere with the outcome of successive entrainments.

### Pheromone gland content

To obtain male sand flies of known age, larval rearing pots were inspected every 2 h, and newly emerged males were transferred to nylon netting cages (18 x 18 x 18 cm) inside plastic bags. These males were kept under the same temperature, RH and L:D conditions as the colony. Humidity was maintained by placing dampened laboratory filter paper inside the plastic bags and sugar (50% fructose solution) was freely available on a small piece of cotton wool inside the holding cage. Males were selected for experimentation at known ages; 0–2, 6–8, 12–14, 18–20, 24–26 hours old and 2, 3, 5, 7, 9, 12 and 15 days old.

To determine the pheromone gland content, six individuals (replicates) of each age category were analysed by GC-MS. Males were individually placed in Pasteur pipette ampoules and covered with a drop (ca. 10 ul) of analytical grade hexane (≥ 99% purity, SupraSolv, Merck, Germany). The ampoules were flame-sealed and left for 24 h at room temperature prior to analysis. Subsequently, the ampoules were opened and the solvent was gently evaporated under N_2_ to a volume of ca. 1 ul. The entire sample was then injected into the GC-MS system.

### Pheromone release

Groups of 10 males, which appeared to be healthy and vigorous, were immobilised by cooling in a freezer for 30 s, and transferred from the holding cage into a clean 50 ml r/b glass flask using a battery powered aspirator. Pheromone released from the males was collected from the headspace volatiles using a portable entrainment apparatus (Barry Pye, Kings Walden, Herts, UK). Clean air was pushed through the 50 ml r/b glass flask containing the male sand flies into a glass tube filled with Tenax, an adsorbent polymer (ORBO 402, Sigma-Aldrich Ltd., Dorset, UK). All joints in the apparatus were sealed with Teflon tape and the airflow at the outlet of the Orbo 402 tube was measured accurately with a bubble flow meter and adjusted with a rotameter (GPE Ltd., Leighton Buzzard, UK) to 400 ml min ^-1^. Adsorbent tubes were used only once. The entrainment was done in an insectary at 26–28°C and 60–80% RH under controlled light conditions.

Volatiles adsorbed on the Orbo 402 tubes were eluted in 2 ml of pure analytical grade hexane. These extracts were collected in small, clean glass Pasteur pipette vials and concentrated under a gentle stream of N_2_ to 10 ul, and then 1 ul (1 male equivalent) was injected into the GC-MS system. Peak areas of the main terpene component present in the extracts were compared to those of known amounts of both caryophyllene (40 ng/ul) and 9MGB (40 ng/ul) as external standards. The major terpene peak usually represents more than 90% of the total terpenes present, and behavioural biossays have demonstrated that it is the active component of the extract [35, 36]. Analytical standards (*n*-alkane series C8-C20) of known concentration (10 ng ul ^-1^) were also injected at the begining and end of each analytical session to provide comparative retention time data, an accurate average for the peak areas, and as a check on the GC-MS system performance.

#### Hourly pheromone release

To determine how much pheromone was released each hour, the adsorbent tube was replaced every hour during the 4 h experiment. Preliminary observations had shown that after 4 h only trace amounts of pheromone were released so entrainment was discontinued at that point. The behaviour of males inside the flask was observed every 15 min throughout the first hour and then every hour for the remaining 3 h of the experiment.

### Effect of the presence of conspecific females on pheromone released

To determine the effect of the presence of females on pheromone release, three 4-day old virgin females were placed in the 50 ml r/b flask along with the 10 males (males + females). The females remained with the males throughout the experiment and the Orbo 402 tubes were replaced each hour as previously described.

### Effect of light or dark on pheromone release

To determine if light or dark had an effect on pheromone release we entrained pheromone from males and males + conspecific females in two lighting conditions light (L) or dark (D). Entrainments were carried out on groups of males and males + females. For each light regime 4–6 experimental replicates were carried out.

### Comparison of gland content with pheromone released

The pheromone content of 5-day old males (*n* = 1 male, 3 replicates) was compared with the amount of pheromone released during 24 h by 5-day old males (12:12 LD) (*n* = 10 males, 3 replicates) for each of the three chemotypes studied. The mortality within the entrainment glass r/b flask varied between 10% to 20%.

### Statistical analysis

A Kruskal-Wallis non-parametric ANOVA was used to compare the amount of pheromone: 1) extracted from the glands of individual males of the three chemotypes at different ages, 2) extracted from 5-day old males with the amount of pheromone released during 24 h by 4–5 day old males, 3) released during each hour of the 4 h period by 4–5 day old males and males + conspecific females, under the two light regimes for each of the three chemotypes. Results are expressed as mean ± standard error (*X* ± SE) ng male ^-1^ hour ^-1^. All statistical analyses were done using SPSS (v15.0, SPSS Inc.) software. Alpha was set at *P* < 0.05.

## Results

### GC-MS analysis of pheromone gland contents and headspace volatiles

**Fig 1:**
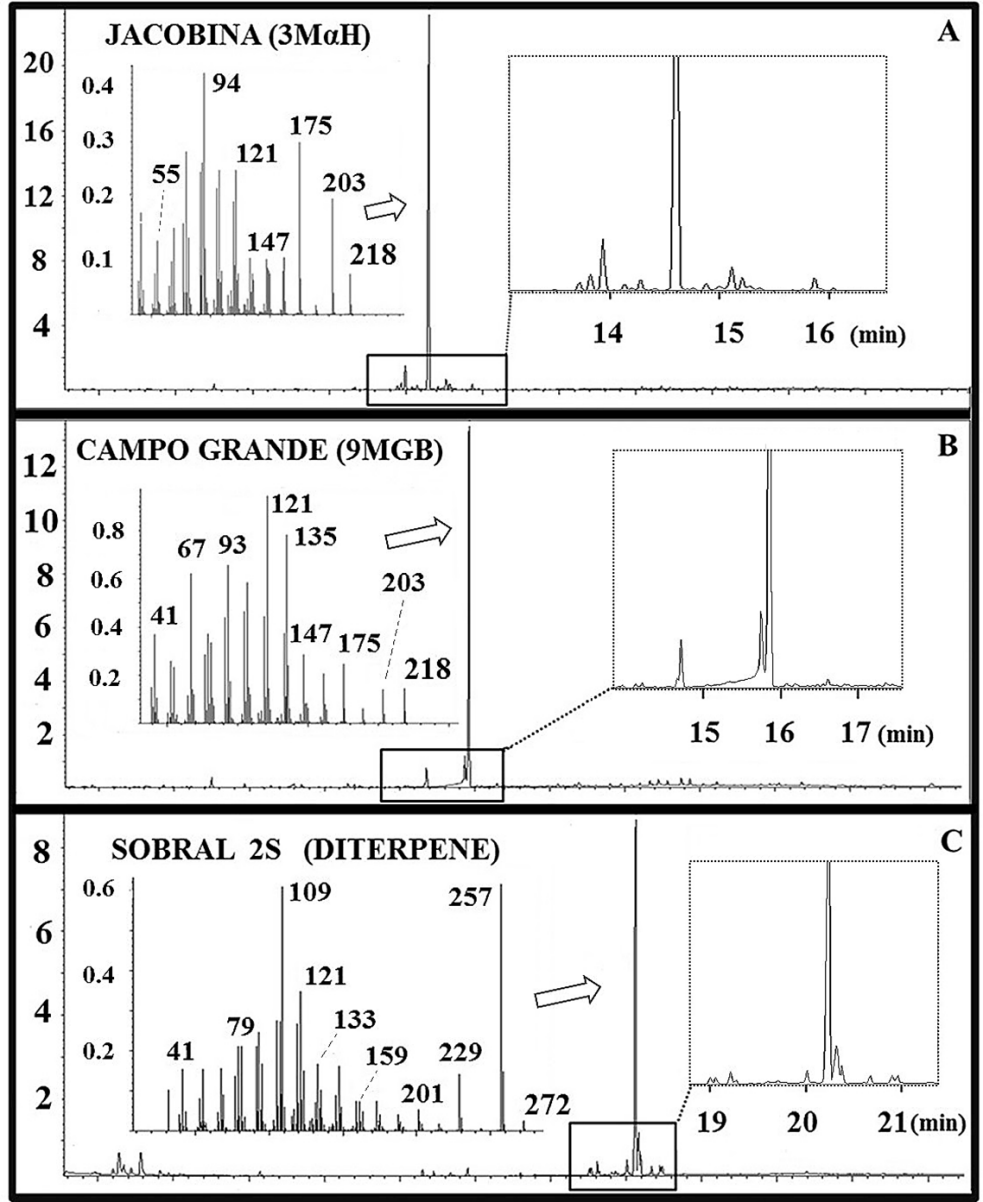
GC-MS analysis of the sex-aggregation pheromone gland extracts of *L*. *longipalpis* males. The total ion chromatogram of each chemotype with mass spectrum of the major pheromone component is shown. **(A)** Jacobina male (3MαH), **(B)** Campo Grande male (9MGB), and **(C)** Sobral 2S male (diterpene).

GC-MS analysis confirmed that the retention times (Rt) of the major peaks of each of the chemotypes were 14.58 min (3MαH, Jacobina), 15.82 min (9MGB, Campo Grande) and 20.17 min (diterpene, Sobral 2S) (Fig 1). Retention time and mass spectral data allowed us to accurately identify the relevant pheromone peak in both pheromone gland and headspace extract chromatograms even when they were present in trace amounts.

The GC-MS analysis of the Jacobina male extract showed that up to 12 other terpene compounds were present in minor quantities (four were present in trace amonts only and were unquantifiable), which eluted both before and after the main peak (Fig 1A). In the Campo Grande male extracts, we noticed two small terpene peaks that eluted before the major 9MGB peak (Fig 1B). In the extract of Sobral 2S males seven other minor diterpene components were apparent (Fig 1C). These data are consistent with previous observations from field collected *L*. *longipalpis* and confirmed that the terpene components of the glands had not changed over the time they were kept in a laboratory colony, Jacobina [37], Sobral 2S [24, 35] and Campo Grande [23]. None of the pheromone extracts from any of the three populations contained significant quantities of the predominant pheromone molecule(s) characteristic of any of the other populations.

### Pheromone gland content

**Fig 2:**
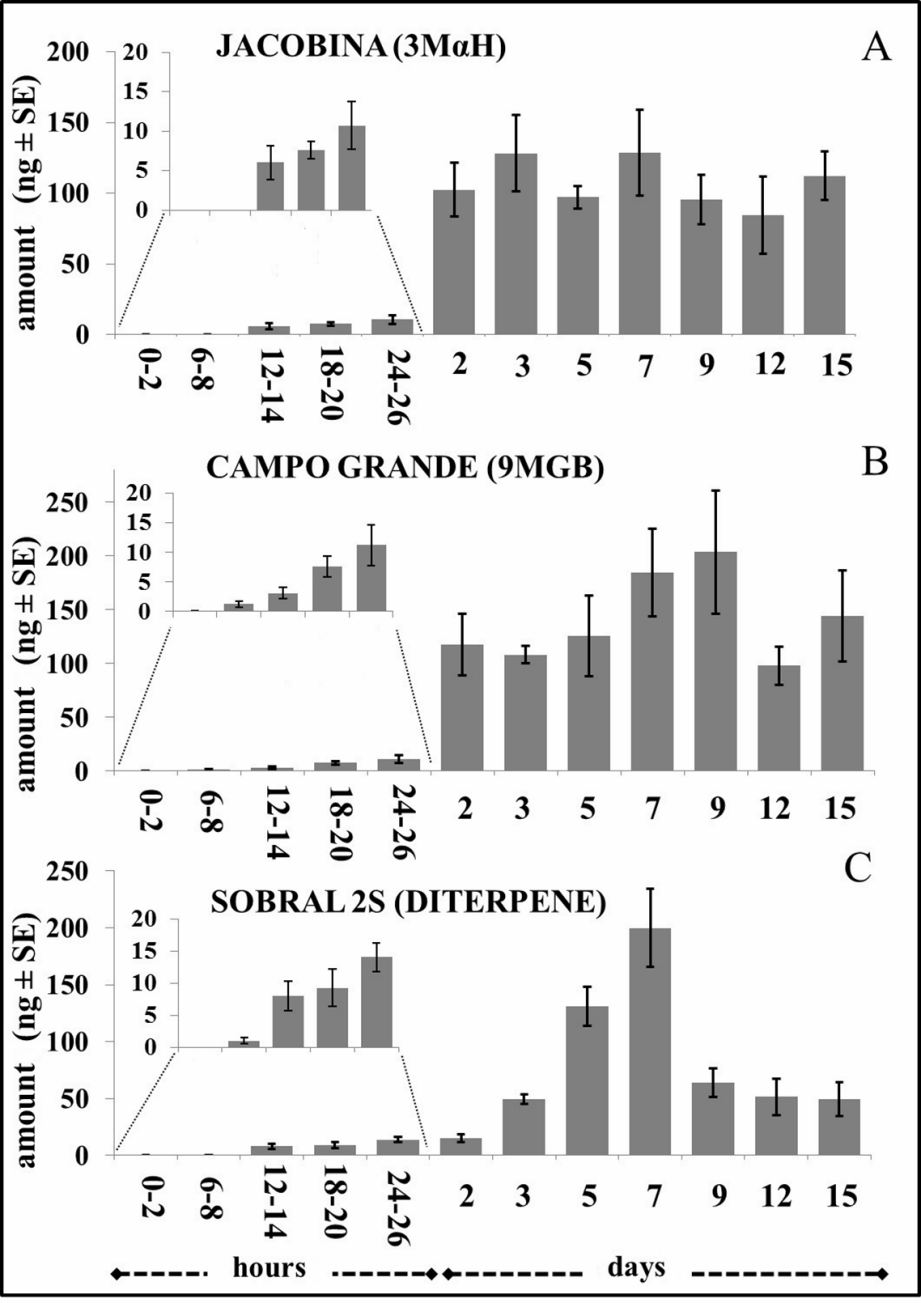
Quantity of sex-aggregation pheromone found in male *L*. *longipalpis* over time for each of the three *L*. *longipalpis* chemotypes. **(A)** Jacobina (3MαH), (**B)** Campo Grande (9MGB), and (**C)** Sobral 2S (diterpene). Each bar represents the mean amount of pheromone present in the gland extract of a single male (ng ± SE) (*n* = 6 replicates for each age interval). Horizontal axis shows time post emergence (hours and days).

Analysis of the pheromone gland content revealed that males of the three chemotypes produced and stored pheromone throughout the 15 days of the experiment, although the amount of pheromone stored varied with age (Fig 2). Traces of pheromone were detected in extracts from individual Campo Grande and Sobral 2S males at 6–8 h post-emergence but were not detected in individual Jacobina males until 12–14 h after emergence (Fig 2).

Each chemotype displayed a distinct pattern of pheromone accumulation over time; generally, pheromone gland content increased for 7–9 days, after which a small reduction in quantity of pheromone was observed in most of the oldest specimens studied. Overall, there was no significant difference in the amount of pheromone present in the glands of the three chemotypes despite large differences in quantities on specific days.

Jacobina gland content (Fig 2A) peaked at 7 days but the amount of pheromone (128.8 ± 30.3 ng) was not significantly different to the other two chemotypes (χ^2^ = 5.81, df = 2, *P* = ns). Campo Grande gland content (Fig 2B) peaked at 9 days (203.5 ± 57.4 ng) and Sobral 2S male’s pheromone gland content (Fig 2C) peaked at 7 days (199.9 ± 34.3 ng).

The biggest increase in pheromone gland content was between 1^st^ and 2^nd^ day old Jacobina males when the average amount of pheromone stored in the glands increased from 10.7 ± 3.0 ng to 102.3 ± 18.9 ng, a 10-fold increase. The Jacobina gland content remained relatively constant for the remainder of the time with a slight dip in pheromone content in older males. A similar increase was observed in Campo Grande males, where the amount of pheromone stored in 2-day old males showed a 10-fold increase (from 11.1 ± 3.4 ng to 117.7 ± 28.7 ng) compared to 1-day old males. Pheromone continued to be produced and content reached a peak at 9 days, after which time it also declined. Pheromone accumulation in the Sobral 2S males was markedly different to the other two chemotypes. There was no increase in the gland content between 1 and 2 days, but the amount of pheromone increased gradually and reached a peak at 7 days, after this time gland content was greatly reduced.

### Behavioural activity

When males were first placed in the 50 ml r/b entrainment flask, they were very active, and appeared to compete with each other to establish space around themselves. This activity occurred both when females were present and absent. Within the first hour of the entrainment, males spent 15–45 min fanning their wings, a behaviour that is associated with pheromone release. Afterwards, they remained mostly motionless for several hours, periodically repositioning themselves within the flask.

### Pheromone release

**Fig 3:**
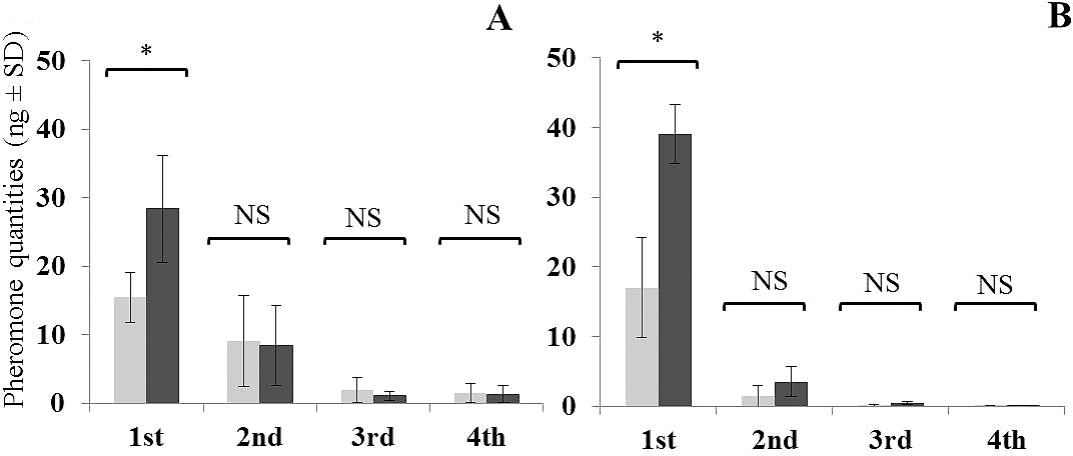
Sex-aggregation pheromone release by Jacobina (3MαH) chemotype, 4–5 day old males during 4 h of continuous air entrainment (ng male^-1^ h^-1^). **(A**) Entrainment carried out in light-only regime with males (light grey) and males + females (dark grey), (**B)** Entrainment carried out in dark-only regime with males (light grey) and males + females (dark grey). * indicates significant differences in quantities entrained between males and males + females for each time period (Kruskal-Wallis test, *P* ≤ 0.05).

**Fig 4:**
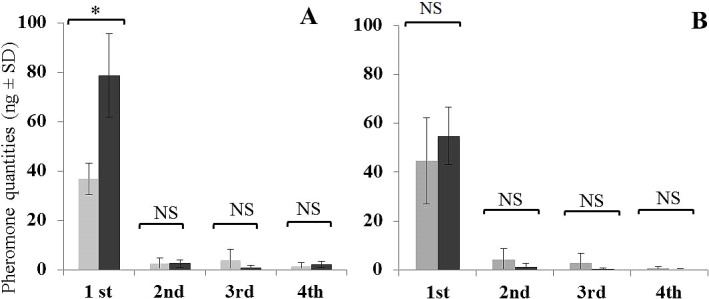
Sex-aggregation pheromone release by Campo Grande (9MGB) chemotype, 4–5 day old males during 4 h of continuous air entrainment (ng male^-1^ h^-1^). **(A)** Entrainment carried out in light-only regime with males (light grey) and males + females (dark grey), (**B)** Entrainment carried out in dark-only regime with males (light grey) and males + females (dark grey). * indicates significant differences in quantities entrained between males and males + females for each time period (Kruskal-Wallis test, *P* ≤ 0.05).

The general pattern of pheromone release for all three chemotypes was that it was greatest during the 1^st^ h of the entrainment. In Jacobina and Campo Grande chemotypes release was significantly reduced during the following 3 h, with release rate values approaching zero in some samples after 2–4 h of continuous entrainment (Figs 3 and 4).

**Fig 5:**
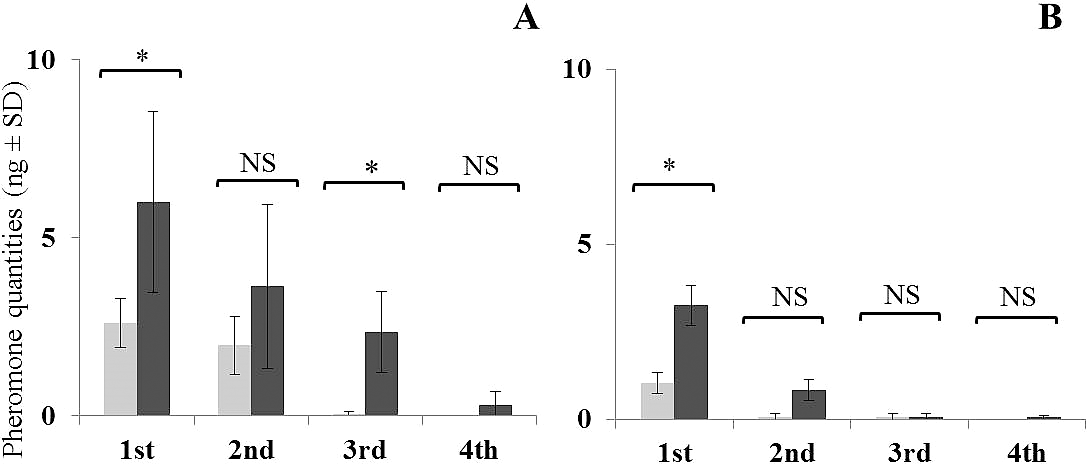
Sex-aggregation pheromone release by Sobral 2S (diterpene) chemotype, 4–5 day old males during 4 h of continuous air entrainment (ng male^-1^ h^-1^). (**A)** Entrainment carried out in light-only regime with males (light grey) and males + females (dark grey), (**B)** Entrainment carried out in dark-only regime with males (light grey) and males + females (dark grey). * indicates significant differences in quantities entrained between males and males + females for each time period (Kruskal-Wallis test, *P* ≤ 0.05).

The amount of pheromone released by the Sobral 2S chemotype males was much lower than for the other chemotypes and the decrease in release over time was more gradual (Fig 5). Nevertheless, this hourly pattern of pheromone release was observed in all three chemotypes in all experimental regimes, both L and D regimes, and in male and male + female entrainments.

### Presence of females

Males always released more pheromone when females were present during the 1^st^ h of the entrainment for all three chemotypes. However, after the 1^st^ h, the presence of females made no significant difference to the release of pheromone for either the Jacobina or Campo Grande chemotypes (Figs 3 and 4). By contrast, Sobral 2S males in the presence of females continued to release more pheromone per hour than males alone although in general without significant differences (Fig 5).

For the Jacobina chemotype, 1.8 times more pheromone was released when females were present under light conditions (χ^2^ = 5.4, df = 1, *P* ≤ 0.05) and 2.3 times when females were present in dark conditions (χ^2^ = 3.0, df = 1, *P* ≤ 0.05) (Fig 3). For the Campo Grande chemotype, 2.1 times more pheromone was released when females were present in light (χ^2^ = 5.4, df = 1, *P* ≤ 0.05), and 1.2 times more when females were present in dark (χ^2^ = 0.3, df = 1, *P* = ns) (Fig 4). For Sobral 2S, 2.3 times more pheromone was released when females were present in light (χ^2^ = 5.7, df = 1, *P* ≤ 0.05) and 3.2 times more when females were present in dark (χ^2^ = 3.8, df = 1, *P* ≤ 0.05) (Fig 5).

### Pheromone release under light or dark conditions

Light conditions appeared to have little effect on pheromone release by the Jacobina and Campo Grande chemotypes (Table 1). However, they were important for pheromone release by the Sobral 2S chemotype where males and males + females released more pheromone under light compared to dark conditions (Table 1).

**Table 1:** Sex-aggregation pheromone release per hour by males of three Brazilian *L*. *longipalpis* chemotypes under two different light conditions, either constant light or dark over a 4-hour period.

		Jacobina		Campo Grande		Sobral 2S	
		(3MαH)		(9-MGB)		(diterpene)	
Period of entrainment	Lighting	♂	♂ + ♀	♂	♂ + ♀	♂	♂ + ♀
**1**^**st**^ **hour**	**L**	15.4 ± 3.7^**a**^	28.3 ± 7.8^**A**^	36.7 ± 6.2^**a**^	78.6 ± 16.8^**A**^	2.6 ± 0.6^**a**^	6.0 ± 2.5^**A**^
	**D**	17.0 ± 7.2^**a**^	39.0 ± 4.2^**A**^	44.6 ± 17.6^**a**^	54.8 ± 11.6^**A**^	1.0 ± 0.3^**b**^	3.2 ± 2.5^**A**^
**2**^**nd**^ **hour**	**L**	9.1 ± 7.6^**a**^	8.4 ± 5.8^**A**^	2.5 ± 2.2^**a**^	2.5 ± 1.5^**A**^	1.9 ± 0.8^**a**^	3.6 ± 2.3^**A**^
	**D**	1.4 ± 1.5^**a**^	3.5 ± 2.2^**A**^	4.2 ± 6.6^**a**^	1.3 ± 1.3^**A**^	0.06 ± 0.1^**b**^	0.8 ± 0.4^**A**^
**3**^**rd**^ **hour**	**L**	1.9 ± 1.8^**a**^	0.5 ± 0.6^**A**^	3.7 ± 4.6^**a**^	0.7 ± 2.0^**A**^	0.04 ± 0.08^**a**^	2.3 ± 1.1^**A**^
	**D**	0.06 ± 0.1^**a**^	0.4 ± 0.2^**A**^	2.9 ± 3.9^**a**^	0.3 ± 0.4^**A**^	0.06 ± 0.1^**a**^	0.06 ± 0.1^**B**^
**4**^**th**^ **hour**	**L**	1.4 ± 1.3^**a**^	1.2 ± 1.2^**A**^	1.3 ± 1.6^**a**^	2.0 ± 1.2^**A**^	0 ± 0^**a**^	0.3 ± 0.3^**A**^
	**D**	0.03 ± 0.05^**a**^	0.05 ± 0.07^**A**^	0.6 ± 0.7^**a**^	0.2 ± 0.3^**A**^	0 ± 0^**a**^	0.06 ± 0.05^**A**^

Jacobina: 3MαH = (*1S*,*3S*,*7R*)-3-methyl-α-himachalene, Campo Grande: 9MGB = (*S*)-9-methylgermacrene-B, and Sobral 2S: diterpene. ♂: 4–5 day old males; ♂ + ♀: 4–5 day old males + 4–5 day old virgin females. Light regime (L) and Dark regime (D). Routine maintenance was performed with photoperiod (12:12, L: 7 am to 7 pm and D: 7 pm to 7 am). Experiments were undertaken either at 7 am after initiation of the photophase (L) or 7 pm after the start of the scotophase (D). Pheromone release amount (ng male ^-1^ hour ^-1^ ± SE). Superscript letters (**a,b**) denote significant differences for males between both light regimes (L and D). Superscript letters (**A,B**) denote significant differences for males + females between both light regimes (L and D). (Kruskal-Wallis test, *P* ≤ 0.05).

For males and males + females of both the Jacobina and Campo Grande chemotypes there was no significant difference in the amount of pheromone released for any of the 4 h periods of both light regimes (Table 1). For the Sobral 2S chemotype, males released almost twice as much pheromone during the 1 ^st^ h in light compared to the dark (2.6 ± 0.6 ng vs 1.0 ± 0.3 ng; χ^2^ = 5.0, df = 1, *P* ≤ 0.05). A higher increase was found during the 2 ^nd^ h (1.9 ± 0.8 ng vs 0.06 ± 0.1 ng; χ^2^ = 5.2, df = 1, *P* ≤ 0.05). When females were present there was a significant difference in the pheromone released in light *vs* dark conditions only during the 3^rd^ h of entrainment (2.3 ± 1.1 ng vs 0.06 ± 0.1 ng; χ^2^ = 6.2, df = 1, *P* ≤ 0.05) (Table 1).

### Pheromone release by the different chemotypes

During the 1^st^ h of entrainment, a significant difference in the average amount of pheromone released by the males and males + females was observed between the three chemotypes. Campo Grande males released the greatest quantities of pheromone, followed by Jacobina males and then Sobral 2S males.

The average pheromone release of individual, 4–5 day old males was significantly different for each of the three chemotypes (χ^2^ = 22.6, df = 2, *P* ≤ 0.001). Campo Grande males released 40.3 ± 12.6 ng, Jacobina chemotype males released 16.0 ± 4.8 ng and Sobral 2S chemotype males 2.0 ± 0.9 ng.

The average amount of pheromone released by 4–5 day old males kept with females was also significantly different for each of the three chemotypes (χ^2^ = 15.1, df = 2, *P* ≤ 0.001). As before, the Campo Grande chemotype males released more pheromone (66.6 ± 17.7 ng), followed by the Jacobina chemotype males (31.9 ± 8.4 ng) and the Sobral 2S chemotype (4.8 ± 1.3 ng).

### Pheromone release compared to gland content

After 24 h of entrainment, 5-day old Campo Grande males had released 92.6 ± 32.1 ng of pheromone; the Jacobina males 61.3 ± 37.2 ng and the Sobral 2S males 90.9 ± 38.9 ng. Five days old, Campo Grande males had 125.5 ± 37.6 ng stored in their pheromone glands, Jacobina males 97.1 ± 7.8 ng and Sobral 2S 131.0 ± 16.9 ng. Thus, overall, males had released 73.7%, 63.3% and 67.4% of pheromone relative to their gland contents, respectively.

## Discussion

This study shows that in *L*. *longipalpis*, the content of the pheromone gland, which is likely to be closely related to biosynthesis and release, is influenced by both internal and external factors. The age of commencement of pheromone production varies between members of the complex. The amount of pheromone present in the gland depends on the chemotype, and the age of the male sand fly. Pheromone gland content is not linearly related to age and in both of the methylsesquiterpene-producing chemotypes (Jacobina and Campo Grande) there is a period of significant increase in gland content, which may reflect either a period of increased pheromone production or improved ability to store the pheromone. In the Sobral 2S chemotype, the gland content increased over a much longer period, from 2 to 7 days. This may reflect changes in the rate of production and/or accumulation of pheromone within the gland; in any case, the pattern is markedly different from the other two chemotypes.

In this study we have also demonstrated that the amount of pheromone released by males depends on several factors, which include whether or not females are present and the activity of the males. The light conditions in which they were held only affected pheromone release in the Sobral 2S chemotype.

Previous studies on the ultrastructure of pheromone glands of *L*. *longipalpis* have shown that male sand flies have structures that could be involved in the storage and subsequent release of sex-aggregation pheromone [38, 39]. These structures appear to develop in synchrony with pheromone gland cell maturation and pheromone content [28]. As we detected the presence of traces of pheromone 6–8 h after eclosion in Campo Grande and Sobral 2S males, and after 12–14 h in the Sobral 2S chemotype, we confirm previous work in which pheromone synthesis was shown to commence 10–14 h post emergence [28, 38, 39]. As technical developments in GC/MS detectors lead to improved sensitivity, it is likely that pheromone production will be seen to start at an even earlier age.

Previous studies have shown that males up to 4-days old [28] or 9-days old [38] have pheromone present in their glands but we have shown in this study that pheromone is present in the glands of 15-day old males. The biological significance of this remains to be determined but clearly suggests that production, storage and potentially release of pheromone is lifelong for males despite younger males having greater success at obtaining matings [39].

The pheromone gland content in the Campo Grande and Jacobina chemotypes increased by 1000% during a 24 h period between 1 and 2 days after emergence. By comparison, the gland content of Sobral 2S chemotype males increased more slowly (300% between day 2 and 3) and started when the males were older. The amounts of pheromone continued to increase in males of all three chemotypes up to 7–9 days old, and thereafter remained constant or gradually declined. This drop, which was particularly noticeable in Sobral 2S males, may partly account for the lack of mating success in older male sand flies [39]. A drop in pheromone gland content with advancing age is common in many groups of insects, such as flies, e.g. *Isoceras sibirica* [40], and moths, e.g. *Ctenopseustis spp*., *Planotortrix octo* and *Epiphyas postvittana* [41]. The differences in pheromone gland content between the three chemotypes and in particular between the methylsesquiterpene producing populations (Jacobina and Campo Grande) and the diterpene population (Sobral 2S) suggest important behavioural or reproductive differences.

A detailed analysis of the terpene composition of some wild populations of *L*. *longipalpis* from Brazil was previously described [24]. The quantity of pheromone found in our Sobral 2S laboratory colony males (132.0 ± 37.9 ng male^-1^ and 199.8 ± 90.9 ng male^-1^ at 5 and 7 days old, respectively) was similar to the amounts found in wild-type mixed-age males (167.9 ± 52.4 ng male^-1^).

The amounts of 9MGB from wild specimens collected from different parts of Brazil varies considerably, e.g. males from Lapinha (Minas Gerais State) produced 116.5 ± 13.5 ng male^-1^ of pheromone which was significantly more than wild type Sobral 1S males (47.8 ± 10.6 ng male^-1^). Our laboratory colonised Campo Grande males produced 125.5 ± 9.6 ng male^-1^ at 5 days and 184.4 ± 10.0 ng male^-1^ at 7 days. This may indicate that wild sand flies contain less sex-aggregation pheromone than laboratory colonised sand flies as a consequence of environmental factors, e.g. temperature, relative humidity, diet, habitat, season and/or physiological stage [42–44]. However, these differences could also occur because the 2 Sobral populations are sympatric whereas the others are allopatric [24, 45, 46]. In addition, these differences may reflect the population substructuring seen across Brazil [12].

Male behaviour in the entrainment flasks was typical of “lekking” males and involved parading, wing flapping, wing fanning and/or wing vibrating, walking forward in short bursts and changing directions, and fighting with other males [39, 47, 48]. Wing flapping and/or fanning has been suggested by some authors to be a way of distributing pheromone [30, 33] and males that fan their wings more than competitor males are more likely to be successful in obtaining a mate [29]. Our results suggest that during this intense activity the males partly deplete their pheromone glands, as there is a notable decline in the amount of pheromone released after the 1^st^ h of entrainment compared to the next 3 h.

After this initial period of activity, males remained largely motionless for several hours. This stage called “quieting” was reported as a possible indication of pheromone communication because the males that were distributed with regular spacing around the flask, may have established a dominance hierarchy [47]. Although the males may use this “quieting” time to replenish pheromone reservoirs after periods of heavy demands, our results suggest that even after 24 h the pheromone in the glands are not completely depleted. It would be useful to measure the amount of pheromone in the glands during and after the resting period to see whether gland content is restored and how long the recovery period is.

More pheromone was released by males of all three chemotypes when conspecific females were present. This was most noticeable during the 1^st^ h when increases of between 15 and 300% were observed. When both sexes were held together, an unquantified increase in male activity including wing fanning and other behaviours were observed, which may account for the greater release of pheromone.

Overall, our study showed that light had little or no effect on pheromone release from males of the Jacobina and Campo Grande chemotypes. However, Sobral 2S males released more pheromone either when alone or with females in light conditions compared to dark. Whether or not the light regime also influences pheromone production by the Sobral 2S chemotype remains to be determined.

In the future it will be interesting to determine the role of other factors on pheromone gland content and release. Studies on other insects have shown that the density and numbers of males and male diet cause changes in the sexual signalling and mating behaviour [49, 50]. In *L*. *longipalpis* other factors such as body size [39] and gland or tergite width [29] have been reported to have no effect. From these studies it is clear that pheromone gland content and release are dynamic and responsive processes.

The work presented here is the first attempt to provide a comparative analysis of the sex-aggregation pheromone gland content and factors that might influence the release of the pheromone of the three most widespread chemotypes of *L*. *longipalpis* from Brazil. Although this study was conducted under laboratory conditions on only one population of each of the chemotypes, it is clear that a number of factors can influence pheromone release and that it is a dynamic and not a passive process. We have shown that the presence of females and light conditions influence pheromone release. The observations suggest that significant behavioural, biosynthetic and ecological differences between the three chemotypes and in particular between the methylsesquiterpene chemotypes (Jacobina and Campo Grande) and the diterpene chemotype (Sobral 2S) occurs. It is likely that there are other factors that may also be important but which we have not investigated, for example numbers of males and the presence of host odour on the pheromone gland content. Further work is also needed to identify the circumstances under which males produce and store pheromone and how other factors could positively or negatively impact both of these. In this study we did not investigate the effect of any of these factors on pheromone gland content and it will be interesting in the future to examine these factors also.

An exploration of other aspects of the sex-aggregation pheromones, such as their volatility and the subsequent dispersion of these chemicals, is critical to understanding their behavioural and ecological importance.

We have looked at three different chemotypes of *L*. *longipalpis* and each of these represent a member of the species complex. However, the precise nature of the *L*. *longipalpis* species complex is unclear and there are several contradictory views on the numbers of sibling species and their associations that have recently been reviewed [10].

A better understanding of chemical communication will also be useful for developing synthetic pheromone for control and monitoring applications, e.g. an understanding of differences in rates and patterns of pheromone release by the methylsesquitepene and diterpene producing members of the *L*. *longipalpis* complex may require different approaches to the practical application of these chemicals.

Finally, we believe that sex-aggregation pheromones are a valuable tool for defining members of the *L*. *longipalpis* species complex. The different pheromones represent important differences between members of the complex because they act as a premating isolating barriers. These variations are underpinned by significantly different biosynthesis in males and receptor biology in females. The differences between the chemotypes that this study highlighted are therefore valuable additional supporting evidence to ultimately help define the members of the complex.
